# Counterfactual Processing of Economic Action-Outcome Alternatives in Obsessive-Compulsive Disorder: Further Evidence of Impaired Goal-Directed Behavior

**DOI:** 10.1016/j.biopsych.2013.01.018

**Published:** 2014-04-15

**Authors:** Claire M. Gillan, Sharon Morein-Zamir, Muzaffer Kaser, Naomi A. Fineberg, Akeem Sule, Barbara J. Sahakian, Rudolf N. Cardinal, Trevor W. Robbins

**Affiliations:** 1 Behavioural and Clinical Neuroscience Institute, University of Cambridge, Cambridge, United Kingdom; 2 Department of Psychology, University of Cambridge, Cambridge, United Kingdom; 3 Bahcesehir University, Istanbul, Turkey; 4 Department of Psychiatry, University of Cambridge, Cambridge; 5 Department of Psychiatry, Queen Elizabeth II Hospital, Welwyn Garden City, Hertfordshire; 6 Postgraduate Medical School, University of Hertfordshire, Hatfield; 7 South Essex Partnership Trust, Springhouse, Biggleswade Hospital, Bedfordshire, United Kingdom

**Keywords:** Behavioral neuroscience, decision-making, goal-directed, habit, obsessive-compulsive disorder, regret

## Abstract

**Background:**

Obsessive-compulsive disorder (OCD) is a disorder of automatic, uncontrollable behaviors and obsessive rumination. There is evidence that OCD patients have difficulties performing goal-directed actions, instead exhibiting repetitive stimulus-response habit behaviors. This might result from the excessive formation of stimulus-response habit associations or from an impairment in the ability to use outcome value to guide behavior. We investigated the latter by examining counterfactual decision making, which is the ability to use comparisons of prospective action-outcome scenarios to guide economic choice.

**Methods:**

We tested decision making (forward counterfactual) and affective responses (backward counterfactual) in 20 OCD patients and 20 matched healthy control subjects using an economic choice paradigm that previously revealed attenuation of both the experience and avoidance of counterfactual emotion in schizophrenia patients and patients with orbitofrontal cortex lesions.

**Results:**

The use of counterfactual comparison to guide decision making was diminished in OCD patients, who relied primarily on expected value. Unlike the apathetic affective responses previously shown to accompany this decision style, OCD patients reported increased emotional responsivity to the outcomes of their choices and to the counterfactual comparisons that typify regret and relief.

**Conclusions:**

Obsessive-compulsive disorder patients exhibit a pattern of decision making consistent with a disruption in goal-directed forward modeling, basing decisions instead on the temporally present (and more rational) calculation of expected value. In contrast to this style of decision making, emotional responses in OCD were more extreme and reactive than control subjects. These results are in line with an account of disrupted goal-directed cognitive control in OCD.

People with obsessive-compulsive disorder (OCD) suffer from an irresistible need to perform certain acts to avoid feared consequences. The consequences are typically the subject of intrusive, obsessive thoughts (1). These compulsions are futile acts that, due to the intensity and frequency with which they must be performed, over time become destructive rather than protective. Despite recognizing that these actions are unnecessary and wishing to stop, OCD patients cannot control their behavior fully. They are unable to resist the immediate urge to perform a compulsion, even though the consequences of capitulation are in the long run often worse than the typically transient experience of anxiety (2). This impairment in making action decisions consistent with future goals is thought to result from a disrupted balance between goal-directed action control and habit 3, 4.

It remains to be seen if this deficit results from accelerated (stimulus-response) habit formation in OCD or from a failure to use prospective outcomes to guide action choice. In this experiment, we adopted a computational psychiatry approach (5) to investigate the latter hypothesis, using a mathematical model of economic behavior containing parameters that previously have been shown to engage key brain regions implicated in goal-directed behavior and OCD, the orbitofrontal cortex (OFC), and caudate nucleus 6, 7. We tested if OCD patients and control subjects differ in the extent to which they utilize the comparison of counterfactual action-outcome scenarios in decision making. By using this paradigm, we could assess the contribution of goal-directed control deficits to OCD in a manner independent of stimulus-response habit formation (which typically arises through behavioral repetition).

Economic decision making is not only guided by expected utility, it is heavily influenced by “what if” scenarios that affect emotion 8, 9. Counterfactual thinking occurs when we consider fictive scenarios and compare our current situation to these imagined alternatives. Emotions such as regret are experienced when we engage in upward counterfactual thinking, observing that our current situation is worse than an alternative that could have been, had we acted differently (10). Past experiences of regret cause individuals to adjust their behavior to avoid this emotion, and like goal-directed behavior, this function is mediated by the OFC and the striatum 11, 12, 13, 14. Interestingly, humans typically choose to avoid potential regret, even if it means making an economically suboptimal choice 15, 16.

Patients with lesions to the OFC constitute the archetypal case of counterfactual dysfunction, experiencing attenuated regret and failing to make choices consistent with its avoidance 17, 18. In other experimental studies, OFC-lesion patients typically show little cognitive impairment but consistently fail to consider and learn from negative consequences of actions, preferring immediate reward 19, 20, 21. Schizophrenia, a psychiatric disorder involving orbitofrontal dysfunction (22), is also associated with diminished regret and failure to use anticipated regret to guide future decisions 18, 23, 24. This deficit is thought to be responsible for some goal-directed decision-making problems in the disorder, along with social dysfunction and a lack of emotional accountability for prior actions (25).

Although some decision-making deficits associated with OCD are similar to those seen in schizophrenia and following OFC lesions, such as impairment in inhibitory control over behavior 26, 27, 28, the affective phenotype of obsessions and anxiety distinguish OCD from these disorders in other ways. Obsessive-compulsive disorder patients have an inflated sense of personal responsibility (29), thought to exacerbate the experience of regret (30). In addition, depression and anxiety, which commonly co-occur with OCD (31), are associated with excessive or unrealistic counterfactual thoughts, hypothesized to cause rumination 32, 33, 34, 35, 36. However, it should be noted that one study recently reported attenuated regret in depressed subjects (37).

We hypothesized that OCD patients would exhibit deficient use of counterfactual forward models of action-outcome alternatives in decision making. However, given the considerable affective component to OCD, we hypothesized that, unlike OFC-lesioned patients, affective responses to backward counterfactuals (e.g., the difference between what was won and what could have been won, had they acted differently) would be exaggerated in OCD. To test these hypotheses, we examined OCD patients and matched healthy control subjects on a modified version of the regret task employed by Camille *et al.* (14). We presented choices between two money wheels that varied in expected value and their potential to evoke the extremes of emotion (e.g., regret/relief) at the time of outcome presentation. We examined the contribution of these parameterized variables to choice behavior to test the hypothesis that decision making in OCD would not be sensitive to projected (forward) counterfactual scenarios. Affective responses were recorded, when the outcomes of choice were revealed, to examine the effect of counterfactual comparison on emotional response.

## Methods and Materials

### Participants

Twenty OCD patients and 20 healthy matched control subjects took part in this study on the day of testing. Detailed recruitment and demographic particulars are available in Supplement 1. Groups were matched for gender, age, and verbal IQ, using the National Adult Reading Test (38) (Table 1). Nineteen OCD patients were medicated, predominantly with selective serotonin reuptake inhibitors (SSRIs). As expected, groups differed significantly in terms of OCD symptom severity on the Obsessive-Compulsive Inventory-Revised (39) and Montgomery-Åsberg Depression Rating Scale (MADRS) (40) and in terms of state anxiety and trait anxiety assessed using the State-Trait Anxiety Inventory (41). Patients with depression scores exceeding 12 on the MADRS (a score indicating very few symptoms) were excluded during recruitment. Nevertheless, two patients presented on the day with scores exceeding our cutoff (moderate scores of 22).

**Table 1:** Group Demographics

	Control Subjects	OCD	*F*	*df*	*p*
Age	46.6 (13.5)	44.25 (8.4)	<1	1,37	ns
NART	16.2 (10.75)	15.1 (7.41)	<1	1,37	ns
Y-BOCS Total		21.5 (5.91)			
Obsessions		10.45 (3.83)			
Compulsions		11.05 (3.23)			
MADRS	1.4 (2.09)	7.5 (6.08)	17.99	1,37	<.001
OCI-R	7.05 (5.97)	31.25 (14.03)	48.16	1,37	<.001
STAI-State	28.42 (5.58)	39 (11.75)	12.67	1,37	<.001
STAI-Trait	31.37 (7.21)	55.9 (9.68)	79.41	1,37	<.001

Mean values and standard deviations are presented in parentheses. Questionnaire data from one control subject were lost due to technical error.

MADRS, Montgomery-Åsberg Depression Rating Scale; NART, National Adult Reading Test; ns, not significant; OCD, obsessive-compulsive disorder; OCI-R, Obsessive-Compulsive Inventory-Revised; STAI, State-Trait Anxiety Inventory; Y-BOCS, Yale-Brown Obsessive Compulsive Scale.

### Procedure

**Figure 1:**
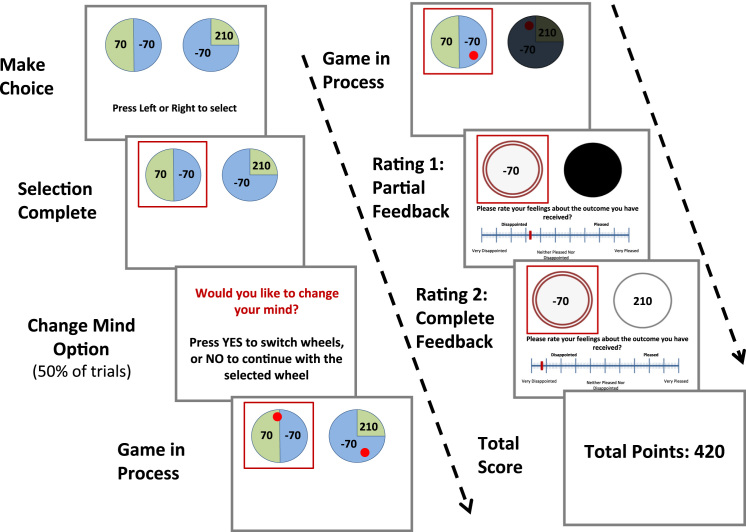
Trial structure of gambling task. The complete list of trials presented to subjects is provided in Supplement 1 (Table S3).

The task employed was based on the gambling paradigms previously used by Camille *et al.* (14) and Chase *et al.* (37). The aim of this task was to maximize points, which would later be converted to money. Subjects were not told the precise relationship between points and money, and all received 4. On each of 80 trials, subjects chose between two wheels that displayed potential gains and losses and their respective probabilities. Each wheel offered two of the following possible outcomes:−210,−70, +70, or +210; each potential outcome’s probability was illustrated by the proportion of the circle occupied by that outcome and could be .25, .5, or .75 (Figure 1). Once subjects had selected a wheel, it was highlighted. On 50% of trials, subjects had the opportunity to change their mind and switch wheels. Once the final selection was made, a ball began to move within each wheel. After 1.5 seconds, the ball stopped on one of the segments in each wheel, determining the value that the subject had won (obtained outcome) and the value the subject could have won had they chosen differently. Before the ball stopped, the nonselected wheel faded to black so that the final result for the nonselected wheel was obscured. It was not possible to extrapolate the final result, as the initial spin path bore no relation to the outcome. At this partial feedback stage, subjects rated their affective response to what they had obtained on a visual analogue scale that ranged from extremely disappointed to extremely pleased. After subjects had completed rating 1, the outcome of the nonselected wheel came into view. Subjects rated their affective responses again at this full feedback stage. Following this, the subjects’ total score was presented on screen.

Ratings taken immediately after the outcome of the subject’s choice was revealed reflected the contribution of a simple chance-based counterfactual (“what if the ball had landed on the other segment of my wheel?”) to emotion. The second emotional rating, which was taken when the outcome of the wheel that was not selected was revealed, reflected the influence of an agency counterfactual of regret/relief (“what if I’d chosen the other wheel?”) on affective response. To reduce the variance between subjects, obtained and nonobtained outcomes were predetermined (i.e., not random) and all subjects received the same trial sequence. Outcomes were closely in line with the presented probabilities and were fair such that on a given trial, the more that avoiding anticipated regret predicted one’s choice, the more likely one was to avoid regret (Figure S1 in Supplement 1).

### Data Analysis

**Affective Responses.** Affective responses were analyzed using linear mixed-effects models. Group (OCD, control) is a fixed-effect factor, trial outcomes (values and counterfactual computations defined below) are continuous fixed-effect predictors, and subject is a random effect factor.

**Figure 2:**
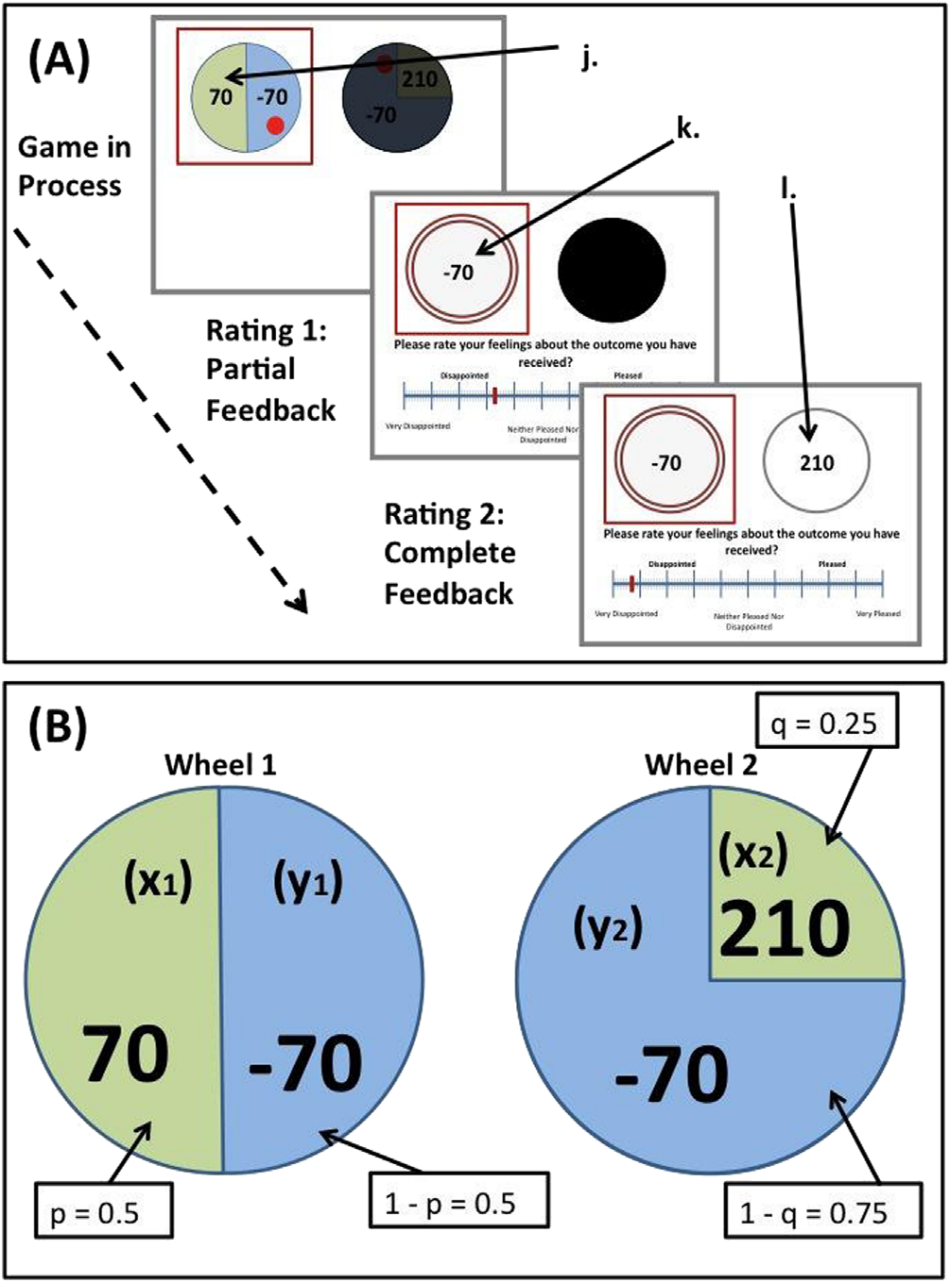
**(A)** Rating regression parameters: obtained outcome = k; chance counterfactual = k−j; agent counterfactual = k−l. **(B)** Decision-making model notation. Outcome x is always the larger (x1>y1 and x2> y2).

We conducted two separate analyses, one for rating 1 (following partial feedback) and one for rating 2 (following complete feedback). For rating 1, we modeled the influence of 1) the value of the obtained outcome; and 2) the chance counterfactual, which is the difference between what was obtained and what could have been obtained had the ball landed in the alternate segment (obtained outcome−nonobtained outcome from that same wheel) (Figure 2). For rating 2, we modeled the influence of 1) the value of the obtained outcome; and 2) the agent counterfactual, which is the difference between what was obtained and what could have been obtained had the subject chosen the other wheel (obtained outcome−nonobtained outcome from the nonselected wheel).

Analyses were performed in R version 2.14.1 (http://www.r-project.org/) using the lme4::lmer function to derive parameter estimates and languageR::pvals.fnc to obtain significance tests for mixed-effects models using Markov chain Monte Carlo simulations (42).

### Decision Making

A model of counterfactual choice behavior similar to that previously used in other variants of this task 11, 17 was fitted to subjects’ wheel selections (equation 4). Specifically, we assessed the influence of three parameters hypothesized to guide decision making: expected value (e: equation 1), variance or risk (v: equation 2), and prospective future regret (r: equation 3).

To construct these models, the possible outcomes and associated probabilities were ascribed the following notation: x_1_ and y_1_ refer to the two possible outcomes of wheel 1 (W_1_), where x_1_>y_1_. Similarly, x_2_ and y_2_ refer the two possible outcomes of wheel 2 (W_2_), with x_2_>y_2_. p and 1-p are the respective probabilities of earning x_1_ and y_1_ and likewise q and 1-q are the respective probabilities associated with earning x_2_ and y_2_ (Figure 2B). Using this notation, the three decision-making parameters (e, v, and r) were calculated. The parameter that maximizes expected value (EV) is denoted e and is calculated by subtracting the EV of W_2_ from the EV of W_1_. If this value is positive, then someone seeking to choose the wheel with the greater EV should choose W_1_. The EV of W_1_ is calculated using: [px_1_ + (1–p)y_1_]. The e parameter is thus defined as:

(1) $$e=EV_{W1}-EV_{W2}=\left[p⁎x_{1}+\left(1-p\right)*y_{1}\right]-\left[q*x_{2}+\left(1-q\right)*y_{2}\right]$$

The avoidance of risk parameter is simply the comparison of the relative variance within each wheel. The equation for the risk associated with W_1_ is p * (x_1_−EV_W1_)^2^ + (1-p)(y_1_−EV_W1_)^2^. A high value indicates that someone seeking to avoid risk should avoid this wheel. The final avoidance of risk parameter (v) is calculated by subtracting the risk associated with W_1_ from that associated with W_2_. If the value is positive, then someone trying to avoid risk should choose wheel 1:

(2) $$\;v=v_{\mathrm{w2}}-v_{\mathrm{w1}}=\left[q*{\left(x_{2}-{\mathrm{EV}}_{\mathrm{W2}}\right)}^{2}+\left(1-q\right){\left(y_{2}-{\mathrm{EV}}_{\mathrm{W2}}\right)}^{2}\right]-\left[p*{\left(x_{1}-{\mathrm{EV}}_{\mathrm{W1}}\right)}^{2}+\left(1-p\right){\left(y_{1}-{\mathrm{EV}}_{\mathrm{W1}}\right)}^{2}\right]$$

The anticipated regret calculation takes into account the magnitude of the difference between the lowest and the highest outcomes across the two wheels (see Supplement 1 for expanded rationale). This calculation is based on the assumption that the greater the difference between what is obtained and what could have been obtained had one chosen differently, the greater the experience of regret/relief. Subjects can minimize the likelihood of experiencing future regret/relief (r) by making the choice that is associated with the smallest differences, i.e., choosing W_1_ if the outcome of the following equation is positive and choosing W_2_ if it is negative:

(3) $$r=\left(y_{1}-x_{2}\right)-\left(y_{2}-x_{1}\right)$$

Using these three parameters, the probability of choosing wheel 1 (Pw1_it_), where t denotes trial (or time) and i denotes individual, is calculated using:

(4) $$P\left(W_{\mathrm{1it}}\right)=1-P\left(W_{\mathrm{2it}}\right)=F\left(v_{\mathrm{it}},r_{\mathrm{it}},e_{\mathrm{it}}\right)$$

F is the inverse logit function, F(θ) = e^θ^(1 + e^θ^) and θ is the logit predicted by the individual values of v, r, and e in the logistic regression. Previous studies using this methodology have included a disappointment parameter (d), which is similar to and indeed correlated with (*r* = .607, *p*<.001), the risk parameter 11, 17, 18. The risk measure (v) was selected, as it is a more accurate measure of within-wheel variance, and unlike d, it is independent of expected value and regret. This parameter allowed us to test for the possibility that OCD patients' behavior was excessively risk-averse. For the purposes of cross-study comparison, however, a model with d instead of v is presented in Supplement 1. None of the main results reported below change with the replacement of v by d.

In keeping with previous studies 11, 17, 18, within-subjects logistic regression analysis was performed using the lme4::lmer function, with the model Choice∼v + r + e + Group:v + Group:r + Group:e + (1|Subject) and subgroup models Choice∼v + r + e + (1|Subject). Choice is a binary variable, coded 1 for wheel_1_ and 0 for wheel_2_; group is a fixed-effect factor; subject is a random-effect factor; e, v, and r are continuous fixed-effect predictors. Where appropriate, estimated effects from the full model were confirmed with a likelihood ratio test, directly comparing models with and without the term of interest (using the stats::anova function). Logit and inverse logit functions are defined as logit(θ) = ln[θ/(1−θ)] and invlogit(θ) = e^θ^/(e^θ^ + 1), such that invlogit[logit(θ)] = θ. Pearson’s product moment correlations were computed between symptom severity ratings in the OCD group and the slope of predictors from the affective rating and decision-making models. A one-way analysis of variance was used to test if OCD patients were more prone to switching wheels than control subjects. Results were considered significant with *p* < .05.

## Results

### Affect Ratings with Partial Feedback

**Figure 3:**
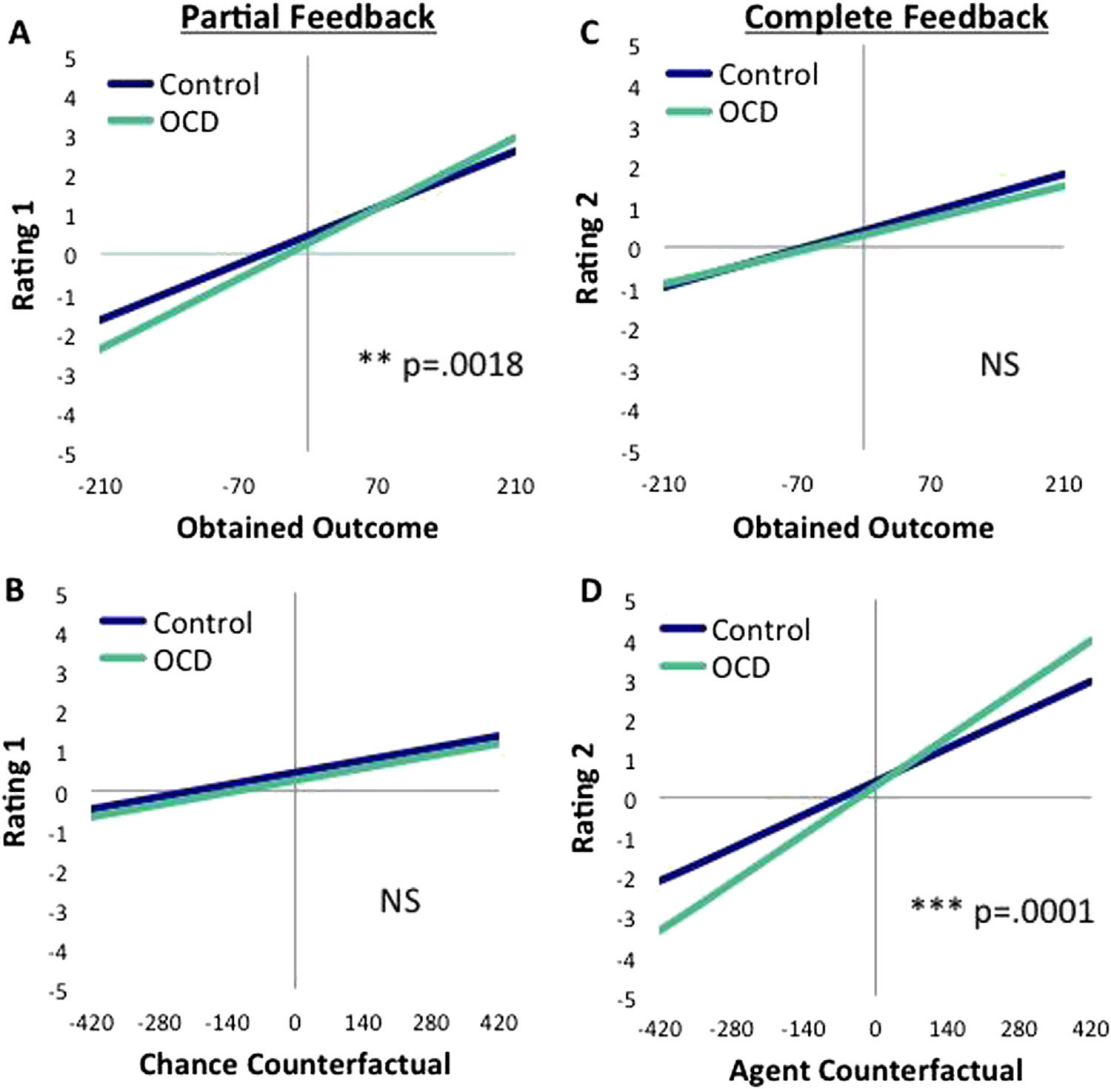
Obsessive-compulsive disorder (OCD) patients show more extreme affective responses to wins/losses and to regret/relief than control subjects. The left panels show the effect of the value of the predictors **(A)** obtained outcome and **(B)** chance counterfactual (obtained outcome−nonobtained outcome from that wheel: k−j in Figure 2A) on rating 1, following partial feedback. The right panels depict the effect of the predictors **(C)** obtained outcome and **(D)** agent counterfactual (obtained outcome−outcome from the nonselected wheel: k−l in Figure 2A) on rating 2, following complete feedback. NS, nonsignificant.

In line with previous data, there was a significant effect for obtained outcome across all participants, *p* = .0001, with more positive ratings with a greater value obtained. The chance counterfactual in the model (the difference between the obtained outcome and what could have been won had the ball landed elsewhere) was also significant, *p* = .0001. There was no main effect of group and OCD patients’ overall ratings were no more positive or negative than control subjects' overall ratings (*p* = .187). However, OCD patients’ emotional responses were more strongly influenced by the obtained result than control subjects (group×obtained outcome, *p* = .0018) (Figure 3A), feeling more pleased when the outcome was good and more disappointed when it was bad. The chance counterfactual did not differentially modulate affective responses between groups, *p* = .992 (Figure 3B). Finally, there was no correlation between OCD symptom severity (Yale-Brown Obsessive Compulsive Scale [Y-BOCS]) and the slope of the obtained outcome parameter, Pearson’s *r*(20) = .131, *p* = .581. In sum, OCD patients showed more extreme affective reactions to the value they won or lost.

### Subjective Affect Ratings with Complete Feedback

At complete feedback, when subjects were shown what they would have won/lost had they acted differently, both the obtained outcome and the agent counterfactual (the difference between the obtained outcome and the outcome of the nonselected wheel) were significant, *p* = .0001, in each case. There was no interaction between group and obtained outcome at this stage, *p* = .2112 (Figure 3C). There was, however, a significant group by agent counterfactual interaction, *p* = .0001 (Figure 3D). Obsessive-compulsive disorder patients’ affective ratings were more strongly influenced by the agent counterfactual relative to healthy control subjects, with the rating more extreme when this difference was either positive or negative. There was no significant correlation between overall Y-BOCS score and the slope of the agent counterfactual *r*(20) = .1, *p* = .624. To summarize, at the second rating, OCD patients showed greater sensitivity to the regret counterfactual than healthy control subjects and there was no longer a difference between the groups on the influence of the value of the obtained outcome on affective ratings.

### Decision Making

**Figure 4:**
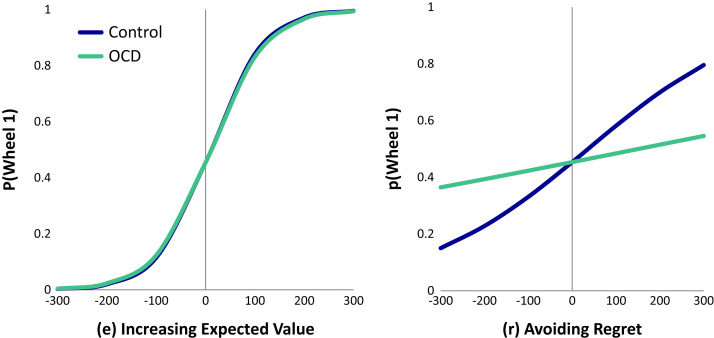
Plots indicating the effect of a given predictor for groups obsessive-compulsive disorder (OCD) and control, according to the regression model, when all other predictors are at zero. For a given predictor X (taking values avoidance of regret [r] and expected value [e]), the ordinate for the regression lines is invlogit(Y), where Y = a + bX; b is the logistic regression coefficient for X from the full model; and a is the intercept.

We tested for group differences in the degree to which the avoidance of regret and risk and the promotion of expected value predicted choice behavior. To summarize the main finding, the use of counterfactual comparison of projected action-outcome alternatives, i.e., the avoidance of future regret, was attenuated in OCD patients compared with control subjects, whereas expected value influenced choice equally in both groups (Figure 4; Table 2). This was evidenced by a significant interaction between group and r but no interaction between group and e (Table 2). On further exploration of these effects (by modeling the two groups individually), we observed that anticipated regret was a stronger predictor of wheel choice in control subjects compared with OCD patients (Table 2). Expected value predicted choice behavior in both groups, with patients and control subjects alike showing a tendency to choose wheels based on the likelihood of winning the most points possible. The risk avoidance parameter did not significantly contribute to the model, and the interaction with group and risk was not significant and was therefore removed (Supplement 1). Goodness of fit of the e, r model was determined using Nagelkerke’s *R*^2^, giving *R*^2^ = .303 (43). There was no difference in the overall points earned by each group (OCD: mean [M] = 763, SD = 553; control: M = 770, SD = 778, *F*<1). Additional analyses, including the effect of task experience over time on decision making, are presented in Supplement 1 for the interested reader.

**Table 2:** Model of Choice Behavior Using Binary Logistic Regression with Individual Random Effects

Parameter	Coefficient	Standard Error	Z Value	*p* Value
(A) Choice Model with All Subjects				
Intercept	−.185581	.0986051	−1.507	.132
r	.0051590	.0003671	14.054	<.0001
e	.0185435	.0018828	9.849	<.0001
r * Group	−.0039275	.0004828	−8.135	<.0001
e * Group	.0007678	.0025810	.297	.766
40 subjects, 3200 observations. Log Likelihood:−1774				

(B) Choice Model with OCD Patients				
Intercept	−.2190821	.1175509	−1.864	.0624
r	.0012207	.0003128	3.902	<.0001
e	.0192052	.0017594	10.916	<.0001
20 subjects, 1600 observations. Log Likelihood:−953.9				

(C) Choice Model with Control Subjects				
Intercept	−.0781293	.1614844	−.484	.629
r	.0051934	.0003687	14.087	<.0001
e	.0186984	.0018918	9.884	<.0001
20 subjects, 1600 observations. Log Likelihood:−819.2				

Panel A shows results from the choice model containing parameters avoidance of regret (r) and expected value (e) and their interactions with group. Each coefficient in the full-choice model refers to the change in log odds per unit change in the given predictor. Therefore, the main effects (r and e) refer to control subjects, who are coded group = 0 and do not represent the average of the groups. Panels B and C show results from applying the model to choice behavior of the OCD patients and control subjects separately.e, expected value; OCD, obsessive-compulsive disorder; r, avoidance of regret.

There was no correlation between Y-BOCS scores and the slope of the e (Pearson’s *r* =−.177, *p* = .624) or r (Pearson’s *r* = .07, *p* = .77) parameters in OCD patients. Likewise, there were no significant correlations between the slopes of any decision-making and affective parameters, indicating that emotional reactivity was unrelated to emotional decision making in OCD, e.g., the degree to which regret influenced affective rating did not correlate with the influence of avoidance of regret on wheel choice, Pearson’s *r*(20) =−.307, *p* = .197. There was no effect of verbal IQ on the use of expected value, Pearson’s *r*(40) =−.212, *p* = .37, or avoidance of regret, Pearson’s *r* =−.175, *p* = .461, to guide choice, and no correlation between IQ and Y-BOCS, *r* = .065, *p* = .786. There were no correlations between depressive symptom severity scores and coefficients from the aforementioned models in the OCD group, all *p*s>.31. We repeated our modeling analyses excluding the two patients presenting with high MADRS scores on the day of testing and the results were unaffected.

## Discussion

Using a mathematical model of choice, we found that the use of counterfactual comparisons of action-outcome alternatives to guide decision making is diminished in OCD patients. This deficit is reminiscent of that found in patients with OFC lesions and schizophrenia. These patients not only fail to avoid future regret but also have a blunted experience of regret consistent with the notion that these disorders have a general failure to compute counterfactuals 17, 18. In contrast, we found that OCD patients’ emotional responses to regret counterfactuals were even more extreme than those of healthy control subjects. When the outcome of the wheel that they had not selected was revealed, OCD patients’ affective ratings were more strongly influenced by the agent counterfactual, i.e., the difference between what was won and what could have been won had they chosen differently. Therefore, unlike OFC-lesioned and schizophrenia patients, OCD patients actually feel the pang of regret and the joy of relief more acutely than healthy control subjects. These results suggest that OCD patients may have a specific deficit in using forward models of prospective action-outcome scenarios to guide behavior in a goal-directed manner, while their ability to compare past counterfactual scenarios is enhanced.

Obsessive-compulsive disorder commonly occurs with depression and anxiety (31), and although this study is limited by the lack of a psychiatric control group, previous studies have shown that these disorders are associated with the excessive generation of counterfactuals thought to contribute to anxiety and rumination 33, 34. The observation that OCD patients are particularly sensitive to the experience of regret could suggest that the symptom overlap between these disorders and OCD is attributable to a common excess of backward counterfactual thinking, a possible contributor to obsessive rumination. However, OCD patients also exhibited more extreme emotional responses depending on the basic value of what they had won. This is not a counterfactual comparison; OCD patients were simply more pleased when they won and more disappointed when they lost. Notably, the predictors that had an exaggerated effect on OCD patients’ emotional responses constituted the most recent piece of information presented to subjects on screen, over and above other factors. At rating 1, this was the value of the obtained outcome, and at rating 2, this was the value of the nonobtained outcome from the wheel that was foregone. Therefore, this pattern of affective responses in OCD patients may be better characterized as heightened emotional reactivity rather than counterfactual generation.

Since the observation that lesions to the OFC disrupt both the experience and avoidance of regret (17), a number of neuroimaging studies have examined the role that this region plays in both counterfactually mediated emotion and choice. A range of paradigms and analytic approaches in functional magnetic resonance imaging have provided convergent evidence for the involvement of the OFC in the experience of regret 11, 44, 45, 46. Regret-related neural activity in the striatum (caudate nucleus and putamen) has been observed in tasks involving a choice between action and inaction 13, 46, 47, 48 and may reflect a counterfactual or fictive prediction error that adjusts future behavior in light of counterfactual comparisons (49). The interface of counterfactual emotion and action was assessed more directly by Coricelli *et al.* (11), who observed that regions involved in anticipating emotion and reflecting prior experience of regret were active at the time of choice, suggesting that avoiding regret might involve anticipating the future emotion.

The present study employed mathematical models to investigate the contribution of a number of predictors derived from economic theories of decision making 8, 10 to choice behavior and affective responses in OCD. This approach to decision-making analysis has been previously used in a number of studies using this paradigm 11, 17, 18, facilitating comparison between these clinical populations and allowing us to make inferences regarding the neural basis of counterfactually mediated choice. While these studies analyzed isolated categorical numeric comparisons (ignoring the majority of comparisons) to investigate subjective experience of these counterfactuals, we adopted a continuous strategy using linear regression. This allowed us to examine the complete range of data points and therefore optimize our analysis.

Obsessive-compulsive disorder patients in this study were medicated, predominantly with SSRIs, posing a limitation to interpretation. No studies have directly assessed the contribution of serotonin to regret and its avoidance. However, the literature suggests that SSRI treatment would likely attenuate, not exacerbate, emotional responses 50, 51, 52, 53. Decision making has also been shown to be amenable to serotonergic influence (54); however, no study has directly tested the role of serotonin in counterfactual choice. One study found that serotonin depletion impaired learning about the relationship between past actions and future aversive outcomes (55), suggesting that SSRIs might have the opposite effect than what we have observed. Nevertheless, possible effects of SSRIs on counterfactual decision making awaits direct test.

This study identifies specific decision-making abnormalities in OCD patients that reflect a lack of forward counterfactual comparison between potential action-outcome scenarios. This finding is consistent with the hypothesis that OCD patients have a deficit in goal-directed control over action (4). Interestingly, we cannot exclude the possibility that by taking the optimum long-term strategy based on expected value, OCD patients generated superior long-term forward models to healthy control subjects, electing to experience regret on a trial-by-trial basis in an attempt to avoid the ultimate regret associated with not earning the maximum total points. This account, however, is limited by the fact that OCD patients did not use expected value to a greater extent than control subjects. In addition to this decision-making abnormality, we found that OCD patients were more emotionally reactive to the outcomes of their actions, an effect that was specific to recently presented outcomes. Together, these findings suggest that a parsimonious account of our observations might be a lack of top-down cognitive control in OCD, an overlapping component of impulsivity and compulsivity (56).

A deficit in the use of counterfactual comparison of future outcomes may underlie the stimulus-driven behaviors that OCD patients feel compelled to perform, in spite of the adverse future consequences. This study, however, does not rule out the possibility that OCD patients also have an overactive habit system; rather, it suggests that deficient processing of future goals likely contributes to their tendency toward compulsive habit behavior. Future research should investigate whether this can fully account for compulsivity in OCD or if excessive stimulus-response habit formation is also a contributor. Indeed, a multifactor explanation might mediate the heterogeneity of both symptom presentation and pharmacologic treatment response evident in OCD.

## Footnotes

Appendix A Supplementary material cited in this article is available online at 10.1016/j.biopsych.2013.01.018.
