# Supplementary material for: Counterfactual Processing of Economic Action-Outcome Alternatives in Obsessive-Compulsive Disorder: Further Evidence of Impaired Goal-Directed Behavior
Source: Biol Psychiatry. 2014 Apr 15;75(8):639–46. doi: 10.1016/j.biopsych.2013.01.018 (PMC3988843; doi:10.1016/j.biopsych.2013.01.018)
Supplement: Supplementary file 1 — Supplementary Material [file mmc1.pdf]

Counterfactual Processing of Economic Action-Outcome Alternatives in Obsessive-Compulsive Disorder: Further Evidence of Impaired Goal-Directed Behavior

Supplemental Information

Participants

This study was approved by the Hertfordshire Research Ethics Committee (07/Q0202/10). Patients were recruited via a specialist outpatient OCD clinic and were screened by a consultant psychiatrist (NAF) using a semi-structured clinical interview to ensure that they met the DSM-IV criteria for OCD (1), with a score exceeding 12 on the Yale-Brown Obsessive Compulsive Scale (2) and did not meet the criteria for any other co-morbid Axis I disorders. Subjects with current alcohol/drug abuse, or a history of neurological illness or head injury were excluded. Control participants were subject to the same exclusion criteria as the OCD patients, aside from the presence of an OCD diagnosis. Participants completed two additional unrelated behavioral tasks in a counterbalanced order in the same session. These results have been, or will be, published elsewhere (3).

Nineteen patients were medicated. 18 were taking selective-serotonin reuptake inhibitors (SSRIs) and 1 patient was taking a serotonin-norepinephrine reuptake inhibitor. As an adjunct to their SSRI, 3 patients were taking antipsychotics, 1 was taking a tricyclic antidepressant, and another was taking the anxiolytic buspirone. One patient was receiving an SSRI, a serotonin antagonist and reuptake inhibitor, an antipsychotic and a mood stabilizer. One patient was unmedicated.

Analyses of Change of Mind

The opportunity to change one's mind and switch wheels has been previously shown to exacerbate the regret effect (4). To test whether this was the case presently, we repeated

our analysis of affective responses at Rating 2, this time including the binary factor "opportunity to change mind" (available on 50% of trials) in the model as an interaction term.

Although subjects could change their minds and switch wheels on 50% of trials, very few did so and even then quite infrequently. There were no differences between groups in the rate of switching wheel ($F < 1$) with the mean number of wheel switches being 1.7 (SD = 2.05) and 1.6 (SD = 2.03) out of 40 opportunities, in controls and OCD patients respectively. We included the opportunity to change wheels in the full model for Rating 2 and contrary to previous reports, the change-of-mind opportunity did not exacerbate emotional responses to obtained outcomes, $p = .377$ or regret/relief (agent counterfactual), $p = .681$ and no three-way interactions with group and obtained outcome, $p = .596$ or regret/relief were observed, $p = .968$. The |v| parameter significantly influenced the likelihood of switching wheels ($p = .006$), but there was no effect of r or e on wheel switch behavior, and no group interactions.

Table S1. Original Choice Model with v, r, e

**Parameter**	**Coefficient**	**Standard error**	**Z value**	***p* value**
Intercept	-0.1259	0.1003	-1.255	.209
v	1.327e-6	3.238e-6	0.41	.682
r	5.151e-3	3.673e-4	14.022	<.0001
e	1.853e-2	1.800e-3	9.854	<.0001
v*group	-7.09e-6	4.313e-6	-1.645	.1
r*group	-3.875e-3	4.839e-4	-8.011	<.0001
e*group	8.222e-4	2.587e-3	0.318	.751

40 subjects, 3200 observations. Log Likelihood: -1772
e, expected value; r, anticipated regret; v, variance.

The v parameter did not contribute to the model and was therefore removed.

In addition, we carried out likelihood ratio tests to confirm that v did not significantly contribute to the model. These data are presented in Table S2 below.

Table S2. Likelihood Ratio Tests. Model 1 is the best fit model, Choice ~ r + e + Group:r + Group:e + (1|Subject), Model 2 is Choice ~ v + r + e + Group:r + Group:e + (1|Subject), and Model 3 is Choice ~ v + r + e + + Group:v + Group:r + Group:e + (1|Subject)

	-Log Likelihood	vs. Model 1	vs. Model2
Model 1	1774.2		
Model 2	1773.5	χ^2 = 1.393<br>*p* = .2379, *df* = 1	
Model 3	1772.2	χ^2 = 4.083<br>*p* = .1299, *df* = 1	χ^2 = 2.6895<br>*p* = .101, *df* = 1

e, expected value; r, anticipated regret; v, variance.

Table S3. Full Task Sequence

	Wheel 1					Wheel 2				
Trial	x1	p	y1	1-p	Outcome	x2	q	y2	1-q	Outcome
1	210	0.5	-70	0.5	210	70	0.75	-70	0.25	70
2	-70	0.75	-210	0.25	-210	210	0.25	-210	0.75	-210
3	70	0.75	-210	0.25	70	210	0.25	-70	0.75	-70
4	70	0.5	-70	0.5	70	210	0.5	-210	0.5	210
5	210	0.25	-70	0.75	-70	210	0.5	-210	0.5	-210
6	70	0.5	-210	0.5	70	70	0.25	-70	0.75	-70
7	70	0.75	-70	0.25	70	210	0.5	-70	0.5	210
8	70	0.5	-70	0.5	-70	210	0.5	-210	0.5	210
9	210	0.25	70	0.75	210	210	0.5	-70	0.5	-70
10	210	0.5	70	0.5	210	210	0.75	-210	0.25	210
11	70	0.75	-210	0.25	70	210	0.5	-210	0.5	-210
12	70	0.75	-210	0.25	70	210	0.5	-210	0.5	-210
13	210	0.25	-70	0.75	210	70	0.5	-70	0.5	-70
14	70	0.75	-70	0.25	-70	210	0.25	70	0.75	70
15	-70	0.5	-210	0.5	-210	210	0.25	-210	0.75	210
16	210	0.25	70	0.75	210	210	0.5	-70	0.5	210
17	210	0.25	-210	0.75	210	-70	0.75	-210	0.25	-70
18	-70	0.5	-210	0.5	-210	70	0.25	-210	0.75	70
19	-70	0.5	-210	0.5	-210	70	0.25	-210	0.75	-210
20	70	0.75	-210	0.25	-210	210	0.25	-70	0.75	210
21	-70	0.75	-210	0.25	-70	210	0.25	-210	0.75	-210

	Wheel 1					Wheel 2				
**Trial**	**x1**	**p**	**y1**	**1-p**	**Outcome**	**x2**	**q**	**y2**	**1-q**	**Outcome**
22	210	0.5	70	0.5	70	210	0.75	-210	0.25	210
23	70	0.5	-210	0.5	-210	70	0.25	-70	0.75	-70
24	70	0.5	-70	0.5	70	70	0.75	-210	0.25	70
25	70	0.75	-70	0.25	70	70	0.75	-70	0.25	-70
26	210	0.5	-210	0.5	210	210	0.25	-210	0.75	210
27	210	0.25	-70	0.75	-70	70	0.5	-70	0.5	70
28	70	0.5	-70	0.5	70	70	0.75	-210	0.25	70
29	210	0.25	70	0.75	70	210	0.75	-210	0.25	210
30	210	0.5	70	0.5	210	210	0.75	-70	0.25	210
31	70	0.75	-210	0.25	70	210	0.25	-70	0.75	-70
32	70	0.5	-70	0.5	70	210	0.25	-70	0.75	-70
33	210	0.75	-210	0.25	-210	70	0.25	-210	0.75	-210
34	210	0.5	70	0.5	70	210	0.75	-70	0.25	-70
35	70	0.75	-210	0.25	70	210	0.25	-70	0.75	-70
36	70	0.75	-70	0.25	70	210	0.5	-210	0.5	210
37	70	0.5	-210	0.5	-210	70	0.25	-70	0.75	70
38	-70	0.5	-210	0.5	-70	210	0.25	-210	0.75	-210
39	70	0.75	-70	0.25	-70	210	0.25	-70	0.75	-70
40	70	0.25	-210	0.75	-210	-70	0.5	-210	0.5	-70
41	70	0.5	-70	0.5	70	70	0.75	-210	0.25	-210
42	70	0.5	-70	0.5	-70	210	0.25	-70	0.75	-70
43	210	0.5	-210	0.5	-210	210	0.25	-210	0.75	-210
44	210	0.25	-70	0.75	-70	210	0.5	-210	0.5	210
45	70	0.75	-70	0.25	70	210	0.5	-70	0.5	-70
46	210	0.25	-210	0.75	210	70	0.5	-210	0.5	70
47	210	0.25	-210	0.75	-210	70	0.5	-210	0.5	-210
48	-70	0.75	-210	0.25	-70	210	0.5	-210	0.5	210
49	-70	0.5	-210	0.5	-70	70	0.25	-210	0.75	-210
50	210	0.5	70	0.5	70	210	0.75	-70	0.25	-70
51	210	0.5	70	0.5	210	210	0.75	-70	0.25	-70
52	210	0.5	-210	0.5	-210	210	0.25	-70	0.75	-70
53	210	0.5	-210	0.5	-210	70	0.25	-210	0.75	-210
54	70	0.75	-70	0.25	70	210	0.5	-70	0.5	210
55	210	0.25	70	0.75	70	210	0.5	-70	0.5	210
56	210	0.5	-70	0.5	-70	210	0.75	-210	0.25	-210
57	70	0.75	-210	0.25	-210	210	0.25	-70	0.75	-70
58	-70	0.75	-210	0.25	-70	210	0.25	-210	0.75	-210
59	70	0.5	-70	0.5	-70	70	0.75	-210	0.25	70
60	70	0.75	-210	0.25	-210	70	0.25	-70	0.75	-70
61	210	0.25	-210	0.75	-210	-70	0.5	-210	0.5	-70
62	210	0.75	-210	0.25	210	210	0.25	70	0.75	210
63	210	0.5	70	0.5	210	210	0.75	-210	0.25	-210
64	210	0.25	-210	0.75	-210	210	0.25	-210	0.75	-210
65	70	0.5	-210	0.5	70	70	0.25	-70	0.75	-70

	Wheel 1					Wheel 2				
**Trial**	**x1**	**p**	**y1**	**1-p**	**Outcome**	**x2**	**q**	**y2**	**1-q**	**Outcome**
66	210	0.25	70	0.75	70	210	0.75	-210	0.25	210
67	70	0.75	-70	0.25	-70	210	0.5	-70	0.5	-70
68	210	0.25	-210	0.75	210	70	0.75	-70	0.25	-70
69	210	0.5	70	0.5	210	210	0.5	-210	0.5	-210
70	210	0.25	70	0.75	210	210	0.5	-70	0.5	210
71	70	0.25	-210	0.75	70	70	0.5	-210	0.5	70
72	-70	0.5	-210	0.5	-210	210	0.25	-210	0.75	-210
73	70	0.5	-70	0.5	70	210	0.75	-210	0.25	210
74	210	0.25	-70	0.75	-70	210	0.5	-210	0.5	210
75	70	0.75	-210	0.25	-210	210	0.5	-210	0.5	210
76	210	0.5	70	0.5	70	210	0.75	-210	0.25	210
77	210	0.25	70	0.75	70	210	0.75	-210	0.25	210
78	210	0.75	-210	0.25	210	210	0.5	-70	0.5	210
79	210	0.5	-210	0.5	210	210	0.75	-210	0.25	210
80	70	0.5	-70	0.5	-70	210	0.5	-210	0.5	-210

Subjects were presented with 80 choice trials between two wheels depicting potential gains and their respective probabilities. The outcomes were predetermined to reduce variability in task experience between subjects. There were 16 possible obtained and non-obtained outcome pairs {-210,210; -210,70; -210,-70; -210,-210; -70,210; -70,70; -70,-70; -70,-210; 70,210; 70,70; 70,-70; 70,-210; 210,210; 210,70; 210,-70; 210,-210}. Although predetermined, on average the outcomes adhered closely to the given probabilities, and avoiding regret lead to a reduced regret experience (Figure S1).

Table S4. Predetermined Regret/Relief Outcome Trials

	x1	p	y1	1-p	O	Ox	x2	q	y2	1-q	O	Ox
Relief	210	0.5	-70	0.5	210	-70	70	0.75	-70	0.25	70	-70
	210	0.5	-210	0.5	210	-210	210	0.75	-210	0.25	210	-210
	70	0.25	-210	0.75	70	-210	70	0.5	-210	0.5	70	-210
	210	0.75	-210	0.25	210	-210	210	0.25	70	0.75	210	-70
	70	0.5	-70	0.5	70	-70	210	0.75	-210	0.25	210	-210
	70	0.75	-70	0.25	70	-70	210	0.5	-210	0.5	210	-210
	210	0.75	-210	0.25	210	-210	210	0.5	-70	0.5	210	-70
	210	0.25	-210	0.75	210	-210	70	0.5	-210	0.5	70	-210
	70	0.5	-70	0.5	70	-70	70	0.75	-210	0.25	70	-70
	210	0.5	-210	0.5	210	-210	210	0.25	-210	0.75	210	-210
Regret	70	0.75	-210	0.25	-210	70	210	0.25	-70	0.75	-70	210
	210	0.5	-210	0.5	-210	210	210	0.25	-210	0.75	-210	210
	210	0.25	-210	0.75	-210	210	70	0.5	-210	0.5	-210	70
	70	0.75	-210	0.25	70	70	210	0.25	-70	0.75	-70	210
	210	0.5	-210	0.5	-210	210	70	0.25	-210	0.75	-210	70
	70	0.75	-210	0.25	-210	70	70	0.25	-70	0.75	-70	70
	210	0.5	-70	0.5	-70	210	210	0.75	-210	0.25	-210	210
	210	0.25	-210	0.75	-210	210	210	0.25	-210	0.75	-210	210
	70	0.75	-70	0.25	-70	70	210	0.25	-70	0.75	-70	210
	210	0.75	-210	0.25	-210	210	70	0.25	-210	0.75	-210	70

O = the outcome, Ox = the outcome presented if that wheel was not selected. To ensure that regardless of choice, all subjects experienced a minimum number of regret/relief outcome scenarios, on 10 trials the agent counterfactual was high regardless of the subjects wheel choice, and likewise on 10 trials the agent counterfactual was low, regardless of choice. This was achieved by altering the non-obtained outcome depending on subjects' choice on those trials only.

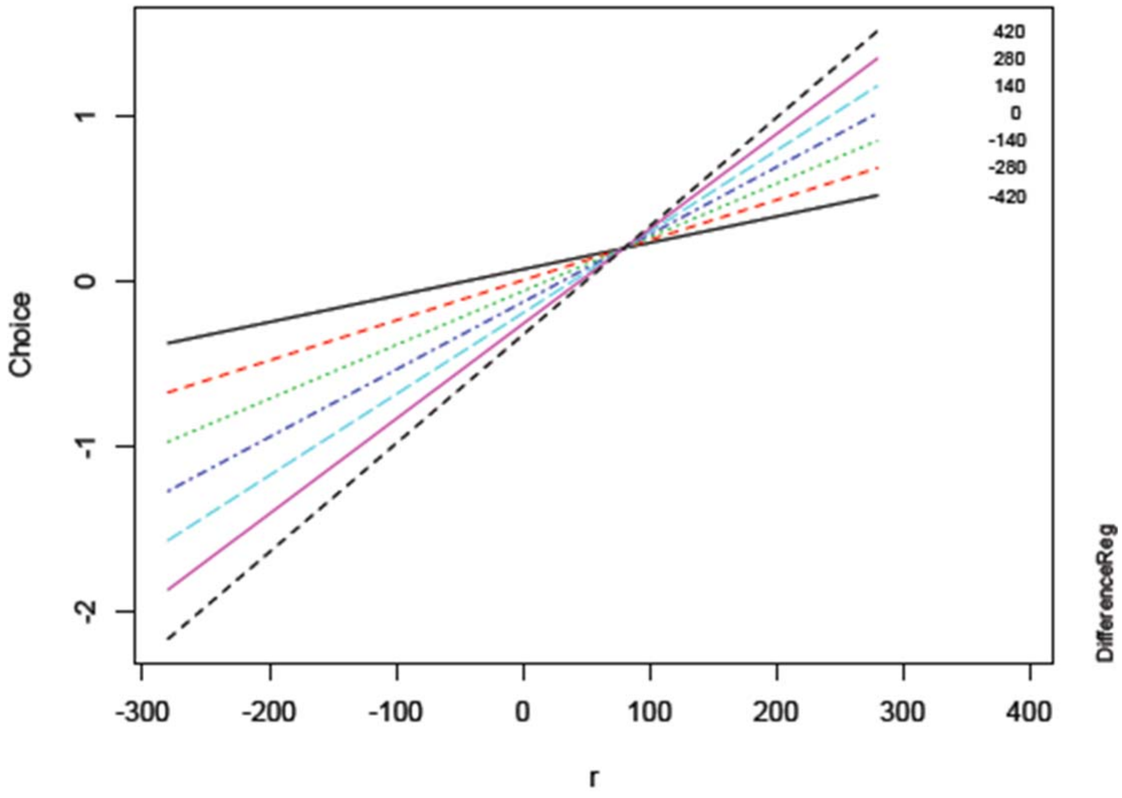

Figure S1. Interaction between r (anticipated regret) and the agent counterfactual. The interaction between r and the agent counterfactual confirms that although some trials were rigged to ensure a minimum number of regret/relief experience trials, electing to avoid regret (steepest line) leads to the least regrettable outcomes (i.e. 420) overall.

The Effect of Experience

We included a trial (1-80) parameter to test for the effect of experience on the use of e, v and r parameters. In line with previous work, we found a significant interaction between trial and r, indicating the subjects became more regret averse over the course of the task (6). Importantly, we noted a significant three-way interaction between group, trial and r (Table S5, Figure S2). Results from the individual model demonstrate that as the experiment progressed, the degree to which anticipated regret influenced wheel choice

increased in control subjects, whereas there was no effect of experience in the OCD patients (Table S5, Panels B, C).

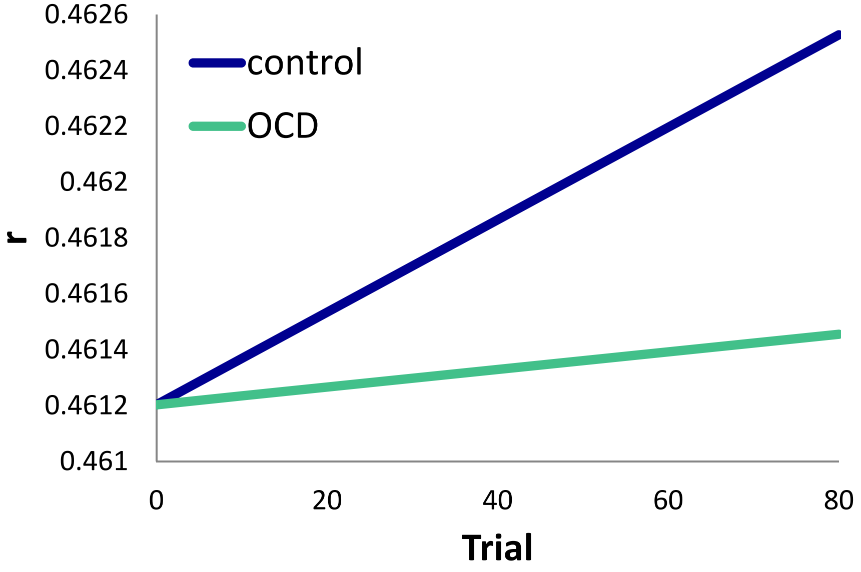

Figure S2. The effect of experience on the use of anticipated regret (r) to guide wheel choice. Control subjects increase their use of this parameter as the task progresses, whereas obsessive-compulsive disorder (OCD) patients do not learn to use this counterfactual comparison.

Table S5. The Effect of Experience on the Use of Anticipated Regret to Guide Wheel Choice

Parameter	Coefficient	Standard error	Z value	*p* value
**(A) Choice model with all subjects**				
Intercept	-0.1555	5.014e-2	-3.101	.00193
r	2.322e-3	7.065e-4	3.286	.00101
e	2.223e-2	4.925e-3	4.537	<.0001
r*group	-1.630e-3	9.495e-4	-1.716	.08614
e*group	-2.642e-3	6.662e-3	-0.397	.69163
r*trial	6.661e-5	1.646e-5	4.046	<.0001
e*trial	-8.698e-5	8.751e-5	-0.994	.32025
r*trial*group	5.392e-5	2.138e-5	-2.522	.01167
e*trial*group	6.218e-5	1.100e-4	0.519	.60406
40 subjects, 3200 observations. Log Likelihood: -1790				
**(B) Choice model with OCD patients**				
Intercept	-0.2167	6.874e-2	-3.152	.00162
r	-5.867e-4	6.414e-4	0.915	.36034
e	2.031e-2	4.534e-3	4.480	<.0001
r*trial	3.645e-4	8.292e-5	-0.440	.66026
e*trial	1.475e-5	1.380e-5	1.056	.29102
20 subjects, 1600 observations. Log Likelihood: -961.4				
**(C) Choice model with control subjects**				
Intercept	-8.085e-2	7.336e-2	-1.102	.27043
r	2.432e-3	7.093e-4	3.429	.0006
e	2.151e-3	4.935e-4	4.358	<.0001
r*trial	6.494e-5	1.653e-5	3.929	<.0001
e*trial	6.942e-5	8.781e-5	-0.791	0.429155
20 subjects, 1600 observations. Log Likelihood: -827.4				

A significant three-way interaction between group, trial and r revealed that while control subjects learned to increasingly avoid regret over time, obsessive-compulsive disorder (OCD) subjects failed to do so.
e, expected value; r, anticipated regret; v, variance.

Modeling Decision-Making

Disappointment (d)

Previous studies have employed a disappointment parameter (d) in the model which takes into account both the likelihood that the worst possible outcome will occur, and the difference between the worst outcome and the best outcome from that same wheel. The

equation for W_1 is $(x_1 - y_1)(1\text{-}p)$. A greater value indicated that someone trying to avoid disappointment should avoid the wheel. The final avoidance of anticipated disappointment parameter (d) is calculated by subtracting anticipated disappointment associated with W_1 from that associated with W_2. If the value is positive, then someone trying to avoid future disappointment should choose wheel 1:

$$d = (x_2 - y_2)(1 - q) - (x_1 - y_1)(1 - p)$$

We thank an anonymous reviewer who observed that this equation differs from e only with respect to the value: $x_2 - x_1$. This is particularly pertinent as parameters are tested for their contribution to the model over and above that accounted for by other parameters. Therefore, rather than comparing the difference between the bad and good outcome within each wheel, assessing the value of this d parameter in large part compares the best outcomes across wheels. For the purposes of comparison with other studies, here we present the analysis using d rather than our alternative, v (Table S4, Figure S3). The main results are unchanged by this substitution.

Table S6. Model of Choice Behavior Using Binary Logistic Regression with Individual Random Effects

Parameter	Coefficient	Standard error	Z value	*p* value
**(A) Choice model with all subjects**				
Intercept	-0.1509	0.1003	-1.504	.1326
d	1.296e-3	7.693e-4	1.65	.099
r	5.780e-3	5.325e-4	10.854	.0001
e	1.717e-2	2.043e-3	8.403	.0001
d*group	-2.303e-3	1.016e-3	-2.267	.0234
r*group	-5.038e-3	6.922e-4	-7.277	.0001
e*group	3.274e-3	2.806e-3	1.167	.2432
40 subjects, 3200 observations. Log Likelihood: -1772				
**(B) Choice model with OCD patients**				
Intercept	-0.1762	0.1213	-1.452	.1464
d	-1.001e-3	6.870e-4	-1.456	.1454
r	7.491e-4	4.492e-4	1.668	.0954
e	2.029e-2	1.926e-3	10.534	.0001
20 subjects, 1600 observations. Log Likelihood: -952.9				
**(C) Choice model with control subjects**				
Intercept	-0.1282	0.1649	-0.778	.437
d	1.247e-3	7.819e-4	1.595	.111
r	5.805e-3	5.380e-4	10.789	.0001
e	1.735e-2	2.058e-3	8.428	.0001
20 subjects, 1600 observations. Log Likelihood: -818				

Panel A shows results from the choice model containing parameters “anticipated disappointment” (d), “anticipated regret” (r) and “expected value” (e), and their interactions with group. Each coefficient in the full choice model refers to the change in log odds per unit change in the given predictor. Therefore the main effects (d, r, and e) refer to controls, who are coded group=0, and do not represent the average of the groups. Panels B and C show results from applying the model to choice behavior of the obsessive-compulsive disorder (OCD) patients and control subjects separately.

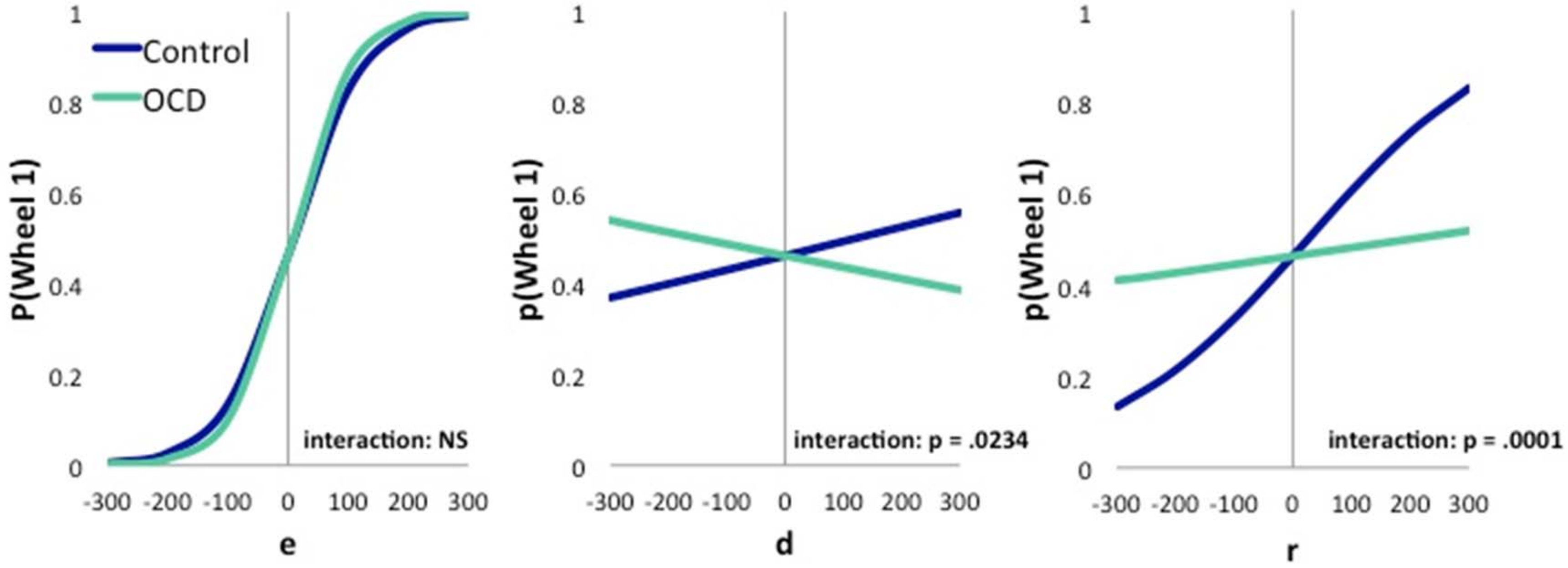

Figure S3. Plots indicating the effect of a given predictor for groups obsessive-compulsive disorder (OCD) and controls, according to the regression model, when all other predictors are at zero. For a given predictor X (taking values d, r, and e), the ordinate for the regression lines is invlogit(Y), where Y = a + bX; b is the logistic regression coefficient for X from the full model, and a is the intercept. e, expected value; d, anticipated disappointment; r, anticipated regret.

Regret

The r equation used in this study ($r' = (y1 - x2) - (y2 - x1) = (x1 - y2) - (x2 - y1) = (x1 + y1) - (x2 + y2)$) differs from that seen in previous publications ($r = |y2 - x1| - |y1 - x2| = |x1 - y2| - | x2 - y1|$) (5-7). In the current dataset, each of these equations produce identical values, owing to a choice of wheels that do not make the optimum choice excessively obvious, so the difference in equation has no bearing on our results; the same is true for these previous studies. However, we suggest that our equation (r′) is theoretically more appropriate when one considers a wider range of possible combinations than is typically presented to subjects (Figure S4).

Regret is the unpleasant perception that one would have done better by selecting a different option (Figure S4A). If one picks wheel 1, one might lose (obtain y1) and regret not having picked wheel 2, where one might have won x2: the potential regret is related to the difference $x2 - y1$. To minimize anticipated regret according to the metric $r = |x1 - y2| - |x2 - y1| = |y2 - x1| - |y1 - x2|$, one should pick wheel 1 when r is positive, and wheel 2 when it is negative ($r < 0$ is shown in A). The lines joining x1 to y2 and x2 to y1 will always cross or touch, since $x1 \geq y1$ and $x2 \geq y2$ by definition. A bold dot is shown on the x value of the better (anticipated-regret-minimizing) option (Figure S4B). A series of comparisons is shown for the metric r and an alternative metric $r' = (x1 - y2) - (x2 - y1)$, with a blob shown on the x value for the preferred wheel in each case. The two metrics make quantitatively, though not qualitatively, different predictions. Take the final (right-most) two choices as an example. For both, the anticipated regret associated with choosing wheel 2 is 4 units (missed gain on wheel 1 minus actual loss on wheel 2). For the penultimate choice, the anticipated regret associated with choosing wheel 1 is 2 units. For the final (right-most) choice, the regret associated with choosing wheel 1 is -2 units (i.e. no regret at all, but

relief). Both metrics predict that wheel 1 would be preferred to wheel 2, but the use of the r metric predicts no difference between the two choices, while the use of r' predicts a stronger preference for wheel 1 (based on regret) in the final choice than in the penultimate choice.

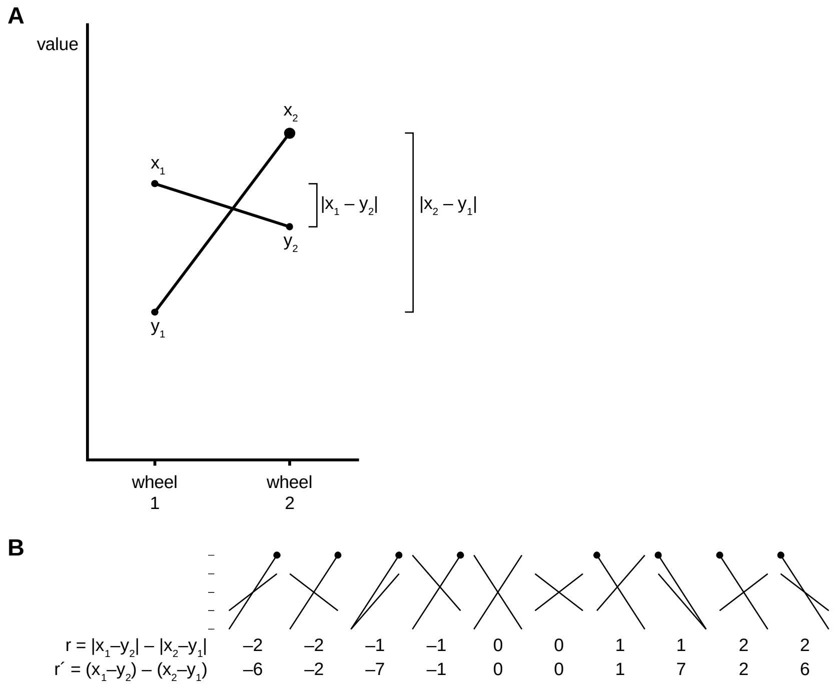

Figure S4. Simulations of r versus r' estimates.

Supplemental References

1. APA (2000): *Diagnostic and Statistical Manual of Mental Disorders*. IV-TR ed. Washington, DC: American Psychiatric Association.

2. Goodman WK, Price LH, Rasmussen SA, Mazure C, Fleischmann RL, Hill CL, *et al.* (1989): The Yale-Brown Obsessive-Compulsive Scale (Y-BOCS): Part 1 Development, use and reliability. *Arch Gen Psychiatry*. 46:1006-1011.

3. Gillan CM, Papmeyer M, Morein-Zamir S, Sahakian BJ, Fineberg NA, Robbins TW, *et al.* (2011): Disruption in the balance between goal-directed behavior and habit learning in obsessive-compulsive disorder. *Am J Psychiatry*. 168:718-726.

4. Camille N, Pironti V, Dodds C, Aitken M, Robbins T, Clark L (2010): Striatal sensitivity to personal responsibility in a regret-based decision-making task. *Cogn Affect Behav Neurosci*. 10:460-469.

5. Camille N, Coricelli G, Sallet J, Pradat-Diehl P, Duhamel JR, Sirigu A (2004): The involvement of the orbitofrontal cortex in the experience of regret. *Science*. 304:1167-1170.

6. Coricelli G, Critchley HD, Joffily M, O'Doherty JP, Sirigu A, Dolan RJ (2005): Regret and its avoidance: a neuroimaging study of choice behavior. *Nat Neurosci*. 8:1255-1262.

7. Larquet M, Coricelli G, Opolczynski G, Thibaut F (2010): Impaired decision making in schizophrenia and orbitofrontal cortex lesion patients. *Schizophr Res*. 116:266-273.
